# Experimental Study on High-Temperature Damage Repair of Concrete by Soybean Urease Induced Carbonate Precipitation

**DOI:** 10.3390/ma15072436

**Published:** 2022-03-25

**Authors:** Hong Wei, Yanan Fan, Lei Sun, Hongxiu Du, Renwang Liang

**Affiliations:** College of Civil Engineering, Taiyuan University of Technology, Taiyuan 030024, China; weihong0052@link.tyut.edu.cn (H.W.); 15135155071@163.com (Y.F.); sunleiayogg@163.com (L.S.)

**Keywords:** soybean urease, concrete, scanning electron microscopy, infrared thermal image, water absorption, compressive strength

## Abstract

In this study, the effects of soybean-urease-induced carbonate precipitation on a high-temperature damage repair of concrete were explored. C50 concrete specimens were exposed to high temperatures from 300 to 600 °C, then cooled to an ambient temperature and repaired by two different methods. The influences of the damage temperature and repair methods on surface film thickness, average infrared temperature increase, water absorption, and compressive strength were investigated. Scanning electron microscopy (SEM) images were carried out to further study the mechanism involved. The results revealed that the white sediments on the surface of the repaired specimens were calcium carbonate (CaCO_3_) and calcium oxalate (CaC_2_O_4_). The surface film thickness reached up to 1.94 mm after repair. The average infrared temperature increase in the repaired specimens at different damage temperatures was averagely reduced by about 80% compared with that before the repair. It showed more obvious repair effects at higher temperatures in water absorption and compressive strength tests; the compressive strength of repaired specimens was 194% higher than that before repairs at 600 °C. A negative pressure method was found to be more effective than an immersion method. This study revealed the utilization of SICP on repairing high-temperature damage of concrete is feasible theoretically.

## 1. Introduction

Concrete structures are the largest type of building structures worldwide, and they will still be the main type of building structures for a long time in the future. Fire is one of the most severe building disasters. The frequency of building fire is very high, and it is easy to cause disastrous consequences. According to the statistics from the Fire and Rescue Department Ministry of Emergency Management of China, there were 109,000 residential fires in 2020, accounting for 43.4% of the total fires, causing 917 deaths and 499 injuries, accounting for 77.5% and 64.4% of the total casualties, respectively.

The fire damage of concrete is mainly due to high temperature. Du [1] expounded on the development form of concrete damage after a fire. The fire damage of concrete results from many factors and is a multi-scale action from the outside to the inside. It is mainly characterized by looseness, peeling, and many V-shaped cracks, which seriously affect the use and safety of concrete structures.

The mechanical properties and durability of concrete are significantly reduced after a fire. Wu et al. [2] prepared C70 and C85 High-Strength Concrete (HSC) specimens and placed them at a high temperature. They found that the HSC specimens explosive spalled when heated to 500 °C, the average compressive strength loss of the two kinds of HSC was 63.2% and 49.2%, respectively, at 500 °C. Gao et al. [3] prepared C20, C40, and C60 concrete specimens to investigate the chloride ion permeability of concrete after high temperatures. They found that the chloride ion migration coefficient of concrete increased significantly with the increase in temperature; the chloride ion migration coefficient of C60 concrete specimens even reached $12.3\times 10^{-12}\;\left(m^{2}/s\right)$ at 800 °C.

In order to deal with high-temperature damage of concrete, some researchers try to improve the fire resistance of concrete. Hossein et al. [4,5] proposed a method. They prepared four mixes containing carpet fibers (0% and 0.5%) and POFA (0% and 20%), then heated them at high temperatures (200, 400, 600, and 800 °C) for 1 h, and then tested the mass loss, residual ultrasonic pulse velocity (UPV) and residual compressive strength of damaged concrete. The results revealed that the addition of carpet fibers to the concrete significantly enhanced the fire resistance and residual compressive strength. At the same time, the explosive spalling behavior of concrete at high temperatures was eliminated. The fire resistance of the concrete mixtures was further enhanced by the inclusion of POFA.

This method can improve the fire resistance of concrete and reduce the damage caused by building fires from the root. Moreover, industrial waste is used, which is in line with the principles of economic and energy efficiency and being environmentally friendly. However, how to repair concrete damages after a fire is not in their research content. Fires still happen frequently, and buildings will still be damaged by fire. If the fire damage of concrete is not repaired timely, external water and corrosive media may penetrate into the damaged concrete, leading to reinforcement corrosion; eventually, the concrete mechanical properties and durability deteriorate rapidly [6]. Therefore, it is imperative to take necessary measures to repair the damaged concrete structures after a fire and restore their performance to ensure safety and prolong their service life.

There are many methods to repair the damage of concrete structures [7], including a replacement method [8], surface sealing method [9], plugging method [10], and electrochemical method [11]. These methods are traditional methods to repair cracks in engineering; they play an important role in practical engineering and reduce the loss caused by cracks. However, these organic materials have many shortcomings: poor adhesion to cement-based materials, poor durability, poor aging resistance, and materials that are harmful to the environment. However, the bionic self-healing method [12] has been developed in recent years, especially the microbial-induced carbonate precipitation (MICP) technique [13,14].

**Figure 1:**
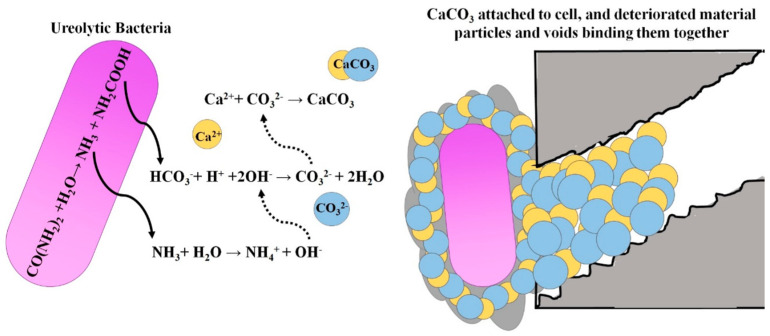
The mechanism of MICP repairing concrete defects.

Some urease-producing bacteria (such as *Sporosarcina pasteurii*) can produce urease through metabolism. Urease can decompose urea in the environment to produce carbonate ions and ammonium ions. Bacteria carry negative charges on their surfaces, and calcium ions in the environment are adsorbed to form calcium carbonate (CaCO_3_) crystals with bacteria as the crystal nucleus. These CaCO_3_ crystals adhere to concrete defects in the liquid environment so as to repair concrete defects. Figure 1 [15] shows the mechanism of MICP repairing concrete defects.

MICP is an environment friendly technique. The research of MICP is currently mainly at the laboratory scale, and there are still several issues in its practical application [16,17]. The cost of large-scale culture of bacteria is high [18], the size and anaerobic or aerobic types of urease-producing bacteria are great obstacles to repair depth [16], the by-products from mineralization remain in concrete defects, negatively impacting long-term performance [13]. In reality, urease is not only produced by bacteria. As early as 1923, Werner [19] found urease in the root nodules of several plants; further, nodules from the white and yellow tree lupin appeared the most active.

Mobley [20] reviewed the high sequence similarity of urease from plants, fungi, and bacteria and concluded that they are variants of the same ancestral protein and are more likely to possess similar tertiary structures and catalytic mechanisms. Therefore, similar to MICP, the microbial culture process can be omitted, and urease can be extracted directly from the urease-containing plants. The extracted urease can be used for CaCO_3_ precipitation to repair high-temperature damage of concrete, which is termed soybean-urease-induced carbonate precipitation (SICP). The mechanism of SICP and MICP are similar. The urease of MICP comes from microbial metabolism, while the urease of SICP is directly extracted from soybean.

Before the SICP technique is applied to repair the fire damage of concrete, it is necessary to find an appropriate method to extract soybean urease. Several researchers studied the extraction of soybean urease and the determination of urease activity. Zhou et al. [21] extracted urease from soybean and determined the K_m_ value of soybean urease as $3.576\times 10^{-2}$ mol/L, the activity of 10 mL urease extract was determined as 26.58 U. Gu [22] compared three methods to determine the urease activity. Compared with colorimetry, ammonia-sensitive electrodes could quickly and accurately determine urease activity, while the pH electrodes could detect the presence or absence of urease activity quickly.

Are the crystals produced by SICP suitable for fire damage repair of concrete? Some researchers studied the crystal structures of CaCO_3_ precipitated by the SICP technique. Yuan et al. [23] studied the effects of several factors on SICP. The results showed that hexahedral CaCO_3_ was produced if the concentration of urease was low, and CaCO_3_ crystals changed to spheres with the increase in urease concentration.

At present, the researches on SICP are mainly in the following fields. Gao et al. [24] used soybean urease to induce CaCO_3_ precipitation to solidify silty soil. In the triaxial consolidated undrained test, the solidified silty soil showed a greater expand response on the stress-strain curve, indicating significantly improved mechanical properties. Gao et al. [25] carried out field tests in Ulan Buh Desert, Ningxia Hui Autonomous Region, China, to evaluate the SICP potential for reducing wind erosion of sandy soil. The results showed that SICP significantly enhanced the surface strength and wind erosion resistance of sandy soil. The optimum concentration and spray amount of consolidating solution (mixed solution of urea and CaCl_2_) was determined as 0.2 mol/L and 4 L/m^2^, respectively.

Research on the numerical parametric analysis of SICP was also carried out. Chen et al. [26] carried out the numerical parametric analysis to assess the effect of EICP enhancement on the hydraulic behavior and slope stability of a sand-geotextile capillary barrier retaining wall during exposure to rainfall with a 100-year return period. The results showed that breakthrough in the sand-geotextile capillary barrier was significantly delayed with increasing concentration of the urea-calcium chloride solutions.

It is obvious that the research on SICP is mainly concentrated in the windbreak, sand fixation, and soil consolidation at present. However, findings on high-temperature damage repair of concrete are sparse. There are many pieces of research on MICP on the repair of concrete defects, and these studies have achieved some results. The mechanisms of MICP and SICP are similar; it is reasonable to believe that SICP can also achieve results in the high-temperature damage repair of concrete. High-temperature damage of concrete is characterized by gradual loosening and cracking from the outside to the inside, and most of the defects are cracks [27]. According to the characteristics of high-temperature damage of concrete, the repair effect of SICP was explored.

In this study, the SICP technique was used to repair high-temperature damage of concrete by two different repair methods. The apparent characteristics, surface film thickness, average infrared temperature increase, water absorption, and compressive strength of concrete were tested to evaluate the repair effect. At the same time, the mechanisms involved were studied by scanning electron microscopy (SEM) imaging and an energy dispersive spectrometer (EDS). The results of this study will provide theoretical bases for applying SICP in repairing high-temperature damage of concrete.

## 2. Materials and Methods

### 2.1. Materials

The raw materials in this study include dried soybean flour (Soyspring Industrial Co., Ltd., Guangxi, China), deionized water, urea analytical reagent (Zhiyuan Chemical Reagent Co., Ltd., Tianjin, China), calcium acetate analytical reagent (Sinopharm Chemical Reagent Co., Ltd., Shanghai, China), P.O 42.5 ordinary Portland cement (Zhihai Concrete Co., Ltd., Shanxi, China), 5–26.5 mm continuously and well-graded crushed stone, IIarea medium sand with fineness modulus of 2.87, and polycarboxylate superplasticizer (Da Shengshi Building Materials Co., Ltd., Guangdong, China) with water-reducing ratio 15–20%.

C50 concrete is often used in buildings and structures, and it was also used in this study. The mix proportion of C50 concrete is shown in Table 1.

### 2.2. Preparation and Treatment of Specimen

#### 2.2.1. Specimen Preparation

For each test, 40 mm cube specimens were cast and cured for 24 h in accordance with GB/T 50081-2019 [28] and GB/T 50082-2009 [29]. Subsequently, the cube specimens were de-molded and kept in a water tank for 28 d; then, the fully saturated concrete specimens were taken out and dried at ambient temperature for 24 h before being tested.

#### 2.2.2. High-Temperature Treatment

**Figure 2:**
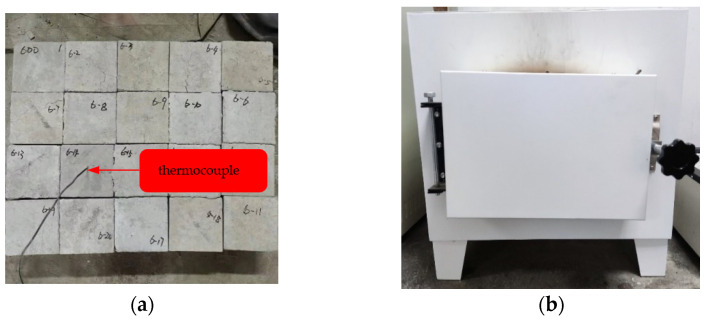
High temperature treatment of concrete specimens. (**a**) Heated concrete specimens. (**b**) Muffle furnace.

The concrete specimens were divided into five groups; they were ambient temperatures, 300 °C, 400 °C, 500 °C, and 600 °C, respectively. There was a thermocouple embedded in the center of one specimen in each group. A muffle furnace was used to heat the specimens. The heating rate was set as 10 °C/min. The temperature in the center of the specimens was shown on a temperature inspection instrument outside the muffle furnace in real-time. When the temperature shown on the temperature inspection instrument reached 300 °C, 400 °C, 500 °C, and 600 °C, respectively, the center of the specimens reached the corresponding temperature, then the muffle furnace was turned off, the furnace door was opened for cooling, as shown in Figure 2.

### 2.3. Test Methods

#### 2.3.1. Extraction of Soybean Urease

**Figure 3:**
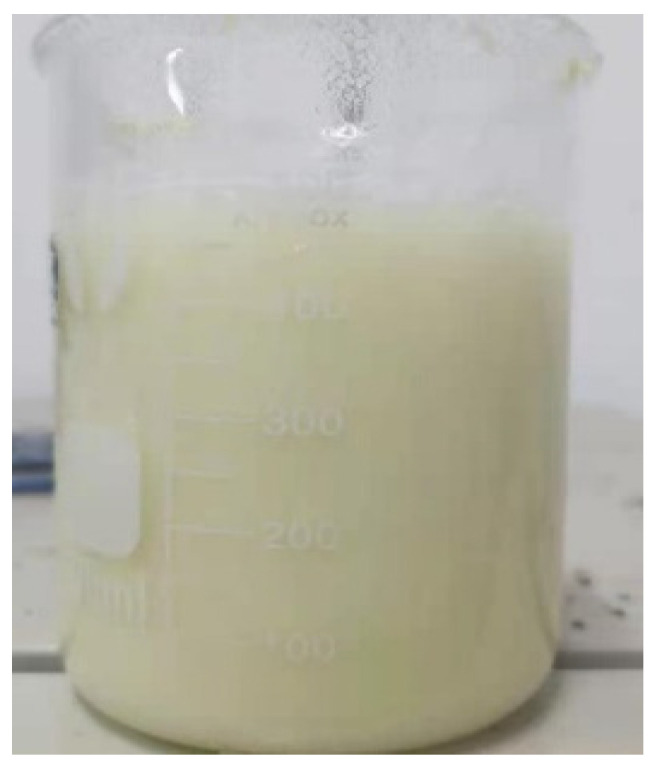
Crude extract solution of soybean urease.

**Figure 4:**
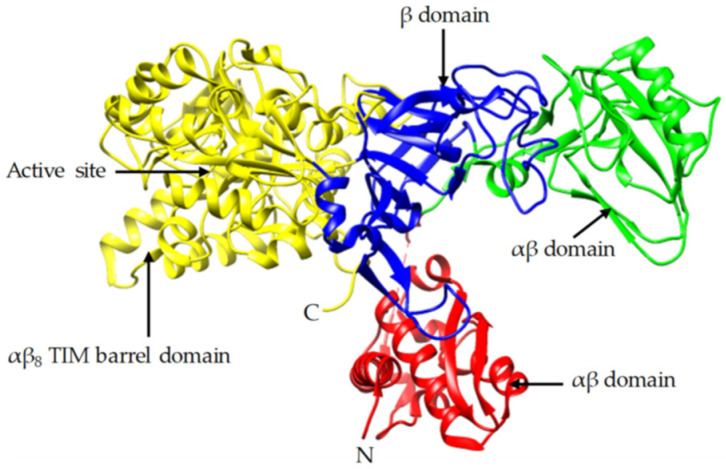
Crystal structure of jack bean urease.

Soybean flour was sifted out with a 100 meshes sieve. It was then added in deionized water, and the mixed solution was stirred evenly and allowed to rest for 2 h. The supernatant was taken out and filtered with gauze. The filtered solution was the crude extract solution of soybean urease. The urease activity of the crude extract solution was tested by the conductivity method proposed by Victoria [14]. From measurement, the urease activity could reach 26.16 U when the concentration of initial soybean flour solution was 200 g/L; this was a high urease activity. In order to decompose urea and repair concrete specimens faster, the concentration of initial soybean flour solution was also 200 g/L in this study. Crude extract solution of soybean urease is shown in Figure 3. Figure 4 [30] shows the structural components of jack bean urease, which is similar to that of soybean urease.

#### 2.3.2. Different Repair Methods

**Figure 5:**
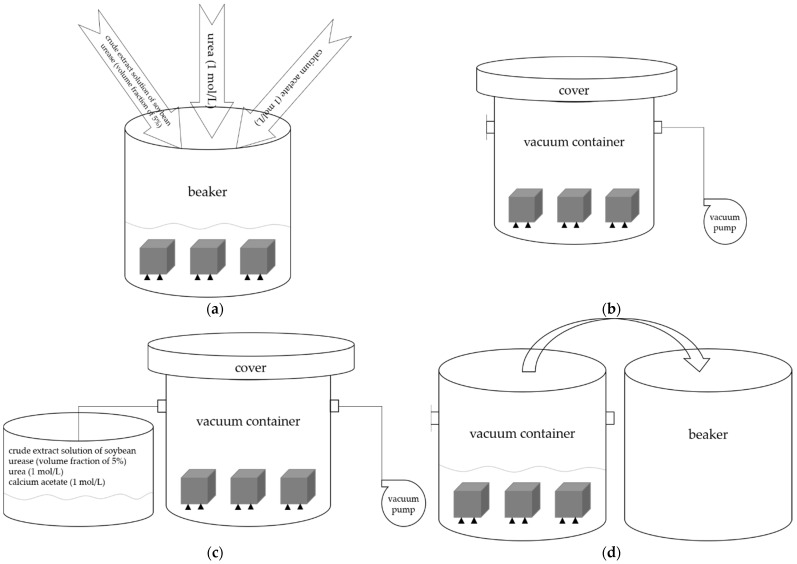
(**a**) The process of immersion method. (**b**–**d**) The process of negative pressure method.

The immersion and negative pressure methods were used to repair concrete specimens damaged by high temperature in this study. Urea and calcium acetate were mixed to prepare 1 mol/L repair solution according to molar mass 1:1 in the two different methods. Crude extract solution of soybean urease was added into the repair solution according to the volume fraction of 5%. The ambient temperature was maintained at 30 °C throughout the process. The specimens were put in a beaker, then the mixed solution of urea, calcium acetate, and crude extract solution of soybean urease was added to the beaker in immersion method. The specimens were subsequently dried after reaching the predetermined repair time, whereas in negative pressure method, specimens were treated with negative pressure refers to the ‘rapid chloride migration coefficient method’ in GB/T 50082-2009 [29]. The specimens were put in a vacuum container, and then the vacuum pump was turned on to reduce the air pressure in the vacuum container to 1–5 kPa within 5 min; this vacuum degree was kept for 3 h with the vacuum pump still running. Then the valve on the vacuum container was opened, and the mixed solution of urea, calcium acetate, and crude extract solution of soybean urease was sucked into the vacuum container to ensure that all specimens were immersed; then, the valve was closed. The vacuum degree was kept constant for another 1 h. Then the specimens in the vacuum container were transferred to a beaker together with the mixed solution. All the other conditions were the same as immersion method. Figure 5a shows the process of immersion method, Figure 5b–d show the process of negative pressure method.

#### 2.3.3. SEM and EDS Experiments

After repair, the white sediments on the surface of repaired specimens along with the repaired specimens themselves were analyzed. The white sediments on the surface of specimens repaired by negative pressure method for 72 h were scraped off to prepare EDS samples. The concrete specimens damaged at 600 °C and repaired by immersion and negative pressure methods for 72 h were cut into two parts, and a small block with repair marks was chiseled by a small hammer to prepare SEM samples. The samples were observed by a TESCAN-LYRA3 scanning electron microscope (Tescan Orsay Holding, a.s., Brno, Czech Republic). The SEM was equipped with an EDS module, which made it possible to analyze the chemical elements and their content in the selected area in real-time.

#### 2.3.4. Surface Film Thickness

The surface of repaired specimens was enlarged by an industrial camera to measure the surface film thickness. The concrete specimens damaged at 600 °C and repaired by immersion and negative pressure methods were taken as research objects. According to Victoria’s [14] formula, it took about 56 h to completely decompose the urea in the mixed solution, so the repair time was set as 24 h, 48 h, and 72 h, which is equivalent to incomplete decomposition, complete decomposition and a period after complete decomposition, respectively.

#### 2.3.5. Infrared Thermal Image

Infrared thermal imaging is a nondestructive test method that transforms the infrared radiation from the targets into visible thermal images, making it possible to analyze the temperature distribution on the surface of objects and to infer the defects on the surface of objects [31]. The infrared thermal images can be processed by MikroSpec (Advanced Energy Industries, Inc., New Jersey, United States) to obtain the average infrared temperature increase; it is a quantization parameter for judging surface defects of objects, and it was used to measure the repair effect of surface defects in this study.

**Figure 6:**
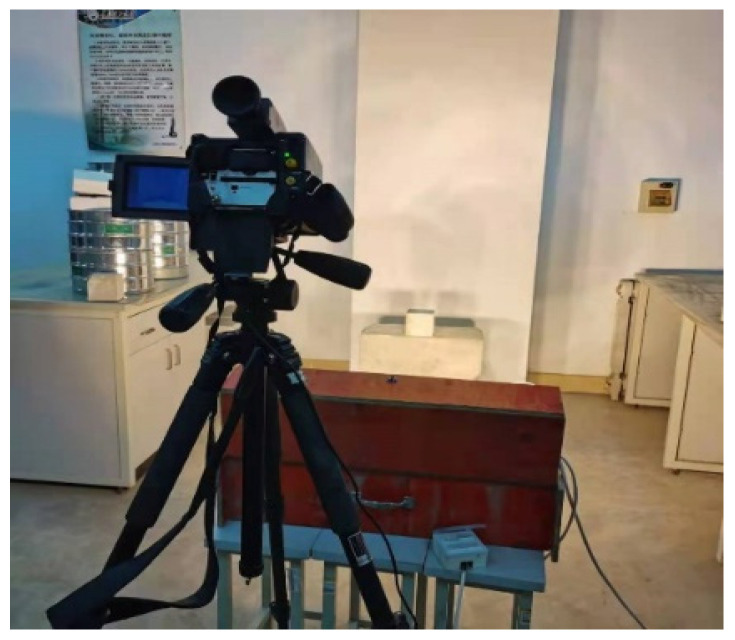
Infrared thermal image test.

The infrared thermal images of concrete specimens before heating, after heating, and after repairs were performed by a TH9100WV (NEC China Co., Ltd., Beijing, China) infrared thermal imager in accordance with GB/T 19870-2018 [32], as shown in Figure 6. The heat sources were infrared bulbs, and the distance between the specimens and infrared bulbs was 1.5 m. We started timing when the bulbs started heating. Infrared thermal images were taken when the specimens began heating, as well as after being heated for 3 min. The specimens before repair and repaired by the immersion and negative pressure methods for 72 h were compared, while the specimens before heating were taken as the control group.

#### 2.3.6. Water Absorption Test

Water absorption is an item of concrete durability reflecting the surface damage degree and the internal compactness of concrete. It was used to measure the repair effect of concrete in this study.

**Figure 7:**
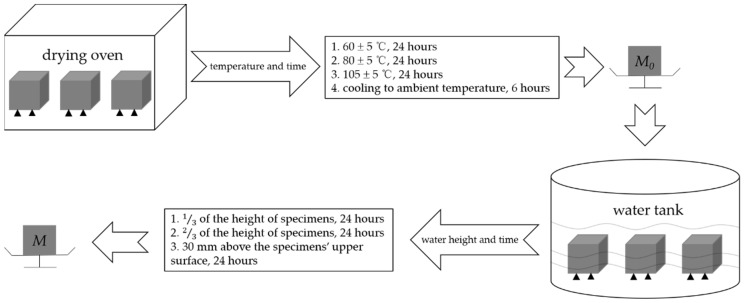
Process of water absorption test.

The concrete specimens before heating, after heating, and after repair were tested for water absorption in accordance with GB/T 11969-2020 [33]. A group of specimens were put into a drying oven. The temperature was first set as (60 ± 5) °C for 24 h, then set as (80 ± 5) °C for another 24 h, and finally set as (105 ± 5) °C until the mass of specimens was kept constant (*M*_0_). After cooling to ambient temperature for 6 h, the specimens were put into a water tank with a temperature of (20 ± 2) °C. Water was added to the water tank; the water height was first kept to 13 of the height of specimens for 24 h, then kept to 23 of the height of specimens for another 24 h, and finally kept to 30 mm above the upper surfaces of specimens for 24 h. After that, the specimens were taken out, wiped off, and weighed the mass (*M*) immediately. The water absorption was calculated according to the formula: $W_{s}=\frac{M-M_{0}}{M_{0}}\times 100\%$. The specimens before repair and repaired by the immersion and negative pressure methods for 72 h were compared. The specimens before heating were taken as the control group. The process of water absorption test is shown in Figure 7.

#### 2.3.7. Compressive Strength Test

Compressive strength is one of the most critical parameters of concrete mechanical properties. The compressive strength of undamaged specimens, damaged specimens, and repaired specimens was compared to reveal the repair effect in this study. The concrete specimens before heating, after heating, and after repair were tested for compressive strength according to GB/T 50081-2019 [28].

#### 2.3.8. Specimens’ Information

Table 2 shows the sample size, repair method, damage temperature, and repair time of specimens for different tests.

## 3. Results and Discussion

### 3.1. Apparent Characteristics

**Figure 8:**
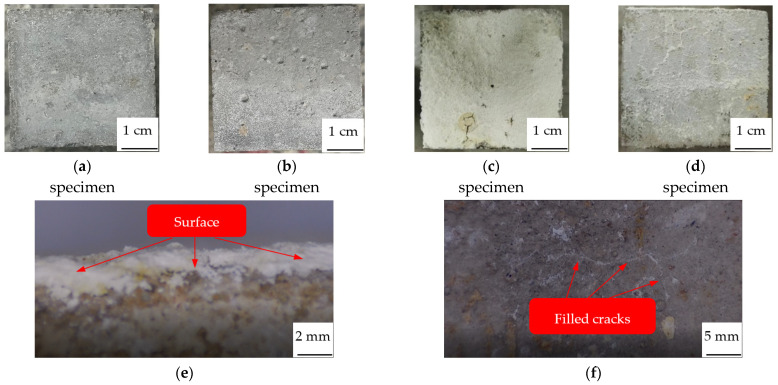
Apparent characteristics of concrete specimen. (**a**) Undamaged. (**b**) Damaged. (**c**) Upper surface of repaired. (**d**) Side of repaired. (**e**) Surface film. (**f**) Repaired cracks.

Figure 8 shows the apparent characteristics of undamaged specimen, damaged specimen and repaired specimen.

Compared with the undamaged specimen (Figure 8a), there are many cracks and pores caused by high temperature; these cracks and pores irregularly distribute on the surface of the damaged specimen (Figure 8b). The cracks connected with each other, the widest reaches 524.57 μm in width, the largest pore reaches 2.26 mm in diameter. However, after repair, the upper surface (Figure 8c) of the specimen is totally covered with two layers of white sediments; cracks and pores are completely filled with the sediments. The particles from the upper layer are large and dispersed, whereas the particles from the bottom layer are compact and continuous with good cohesiveness. These two layers of white sediments will be analyzed in Section 3.2 and Section 3.3. The sides (Figure 8d) of the specimen are also covered with a continuous layer of white sediments but not as thick as the upper surface; cracks and pores are also completely filled with the sediments.

Concrete is a hydrophilic material; when the dry specimen is immersed in the repair solution, it will quickly absorb the repair solution. With the action of negative pressure, the repair solution enters the interior of the specimen, so the white sediments deposit on both the upper surface, the sides, and even on the bottom surface of the concrete specimen. As a consequence, the cracks and pores on the sides are completely filled with white sediments. The repair solution is runny, and gravity plays an important role in the deposition of sediments; more sediments are deposited on the upper surface than on the sides, so the film thickness of the upper surface is thicker than that of the sides. Fan et al. [34] also mentioned the significant stratification phenomenon in her paper, but she has not explained the reason. This may be due to the different crystals of the sediments from two layers; the weight of different crystals is also different, as they will be stratified under the action of gravity, with the heavy crystals on the bottom while the light crystals are on the surface. After all, the upper surface and sides are all immersed in the repair solution, but the sediments on the sides are not stratified, unlike the upper surface.

### 3.2. EDS Analysis

**Figure 9:**
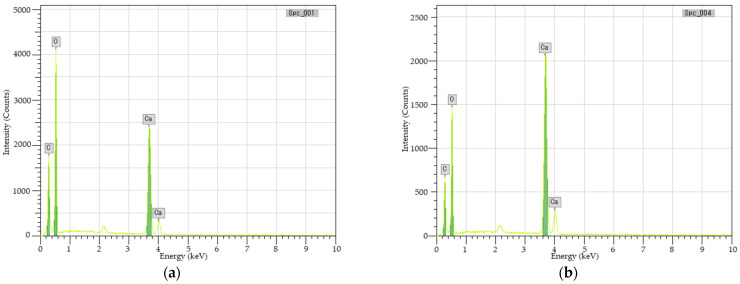
Energy spectrum of the two layers of white sediments. (**a**) upper layer. (**b**) bottom layer.

The white sediments on the upper surface of the specimen were scraped off in layers and analyzed by EDS. Figure 9 shows the energy spectrum of the two layers of white sediments. Table 3 shows their element content.

Figure 8 confirms that whether upper or bottom layer, the white sediments mainly contain C, O, and Ca. Table 3 shows that the molar ratio of C:O:Ca of the upper sediments is approximately 1:4:2, while the bottom sediments are approximately 1:3:1, indicating that the upper sediments may be calcium oxalate (CaC_2_O_4_), the upper sediments may be calcium carbonate (CaCO_3_).

### 3.3. SEM Analysis

**Figure 10:**
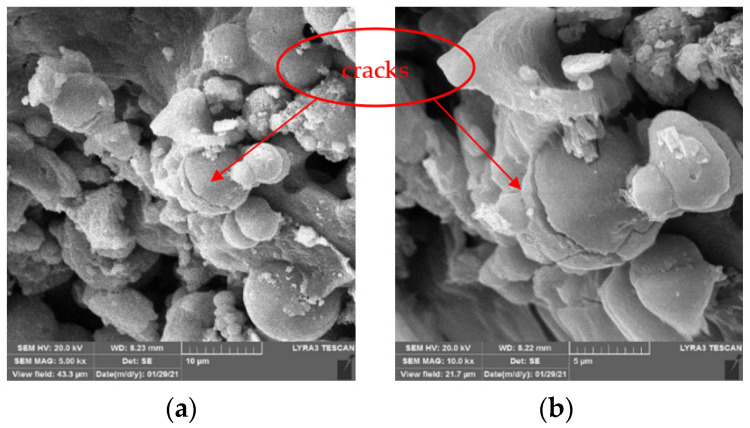
SEM images of CaC_2_O_4_ crystals from upper layer. (**a**) 5 kx. (**b**) 10 kx.

**Figure 11:**
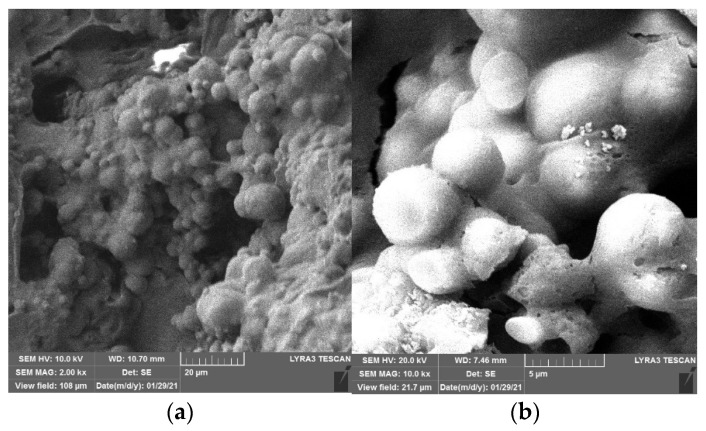
SEM images of CaCO_3_ crystals from bottom layer. (**a**) 2 kx. (**b**) 10 kx.

Figure 10 and Figure 11 show the SEM images of CaC_2_O_4_ crystals from the upper layer and CaCO_3_ crystals from the bottom layer, respectively.

As Figure 10 and Figure 11 show, under the action of soybean urease, urea in the repair solution is decomposed by soybean urease to produce carbonate ions, combining with the calcium ions in the repair solution to form CaC_2_O_4_ and CaCO_3_ crystals. There are obvious differences in morphology and size between CaC_2_O_4_ and CaCO_3_ crystals. The CaCO_3_ crystals from the bottom layer are almost solid spheres with smooth surfaces, and the spheres are uneven in size with a diameter of 5–10 μm. They stacked with each other and arranged closely and continuously. In contrast, the CaC_2_O_4_ crystals from the upper layer are mainly spheres, with some irregularly shaped crystals attached to them. Unlike CaCO_3_ crystals, their surfaces are rough, even cracks distributed on the surfaces of some crystals. The spheres are smaller with a diameter of 2–10 μm, compared with the CaCO_3_ crystals from the bottom layer.

The difference between the morphology and size of CaC_2_O_4_ and CaCO_3_ crystals may be due to the high activity of soybean urease in the early stage of decomposition, which makes the urea in the repair solution quickly decomposed to produce carbonate ions and ammonium ions. At this time, the content of carbonate ions in the repair solution is significantly less than that of calcium ions, so carbonate ions are surrounded and attracted by calcium ions; the crystallization speed on each crystal plane is almost the same, so the crystal morphology is a sphere. Large crystals have a relatively large weight; under the action of gravity, they precipitate on the bottom layer. As decompose time goes by, the decompose rate decreases with the decrease in soybean urease activity. At the same time, the content of calcium ions in the repair solution decreases due to the precipitation of crystals. At this time, the crystallization speed on each crystal plane is different. The growth of crystal morphology is irregular, CaC_2_O_4_ and some irregular crystals are formed, even cracks appear on their surface.

Notably, the crystals generated on the surface of specimens are smaller than the particle size (5–80 μm) obtained by Qian et al. [35]. This is mainly due to the different types of ureases. Qian et al. used the MICP technique and immersion method to form a coating on the surface of concrete; the urease activity of bacteria is higher than that of soybean urease used in this study. In the early stage of decomposition, higher urease activity is advantageous for the formation of larger spherical crystals. However, too large crystals are not easy to enter the micro defects of concrete caused by high temperature. Therefore, the MICP technique used by Qian et al. is not suitable for the high-temperature damage repair of concrete.

**Figure 12:**
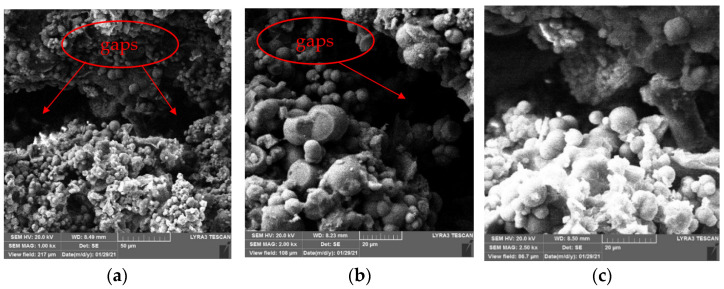
SEM images of specimens repaired by immersion method. (**a**) 1 kx. (**b**) 2 kx. (**c**) 2.5 kx.

**Figure 13:**
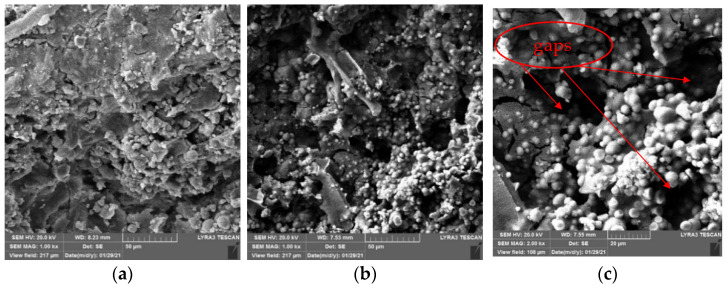
SEM images of specimens repaired by negative pressure method. (**a**) 1 kx. (**b**) 1 kx. (**c**) 2 kx.

Figure 12 and Figure 13 show the SEM images of concrete specimens repaired by immersion and negative pressure methods, respectively.

As Figure 12 shows, in the immersion method, the crystals attached to cement gels inside specimens are mainly spheres with some irregular crystals, but there are still large gaps existing. This is because it only relies on the gravity of the crystals themselves and the permeability of repair solution in immersion method, no external force to promote the repair solution entering concrete specimens along the defects on specimens’ surface, only a small amount of repair solution enters the interior of the specimens. Therefore, few sediments generate inside the specimens, the adhesion between the sediments and cement gels is weak. However, it can be seen from Figure 12 that SICP still changes the internal microstructure of specimens, many crystals attached to cement gels, which makes the internal structure of concrete damaged by high temperature denser. Moreover, the repair solution entering the concrete specimens provides a liquid environment, which promotes the rehydration of the damaged concrete specimens and makes them denser; this may improve their mechanical properties and durability.

It can be seen from Figure 13 that the same as immersion method, the crystals are also mainly spheres in negative pressure method, there are few irregular crystals. Different from that of the immersion method, the connection between crystals and cement gels after negative pressure treatment is closer. There are still gaps but not as large as that of the immersion method, and the particle spacing is smaller. At the same time, the crystals are more evenly attached to cement gels, and the coverage continuity is better. Compared with the immersion method, which only under the action of gravity and weak permeability, more repair solution enters the interior of the specimens due to the action of negative pressure, more sediments generate inside specimens. This is one of the reasons why the negative pressure method can repair high-temperature damage of concrete better than the immersion method.

### 3.4. Surface Film Thickness

**Figure 14:**
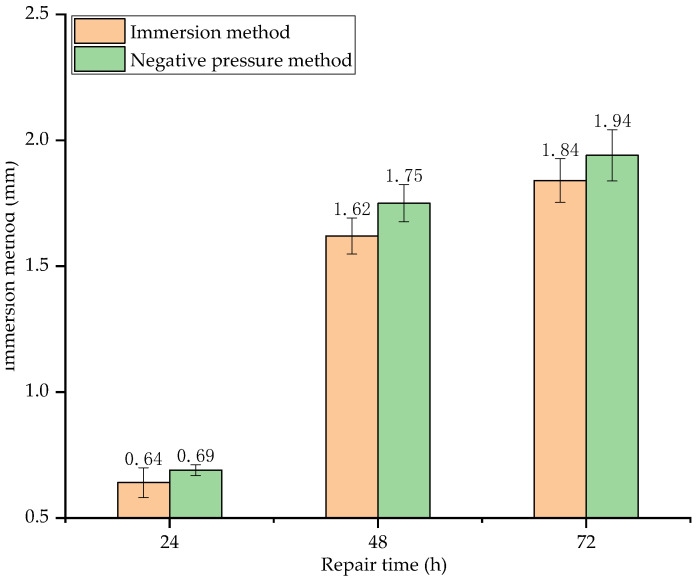
Surface film thickness of specimens repaired by different methods and for different repair time.

Figure 14 shows the surface film thickness of concrete specimens repaired by different methods and for different repair time.

As seen in Figure 14, the surface film thickness increases with repair time. Before 48 h, the surface film thickness increases rapidly. At 48 h, the surface film thickness of the specimens repaired by immersion method and negative pressure method increases by 153.13% and 153.62%, respectively, compared with that of 24 h. After 48 h, the surface film thickness increases slowly. At 72 h, the surface film thickness of the specimens repaired by immersion method and negative pressure method only increases by 13.58% and 10.86%, respectively, compared with that of 48 h. This is because the activity of soybean urease in the early stage of decomposition is very high, so the decomposing rate of urea is also very high. As a consequence, the film thickness increases rapidly. After 48 h, as the soybean urease molecules are wrapped by the generated crystals, the urease concentration in the repair solution decreases, the formation rate of sediments decreases with the decrease in decompose rate of urea. As a consequence, the increase in surface film thickness is not obvious.

As for the surface film thickness of the two different repair methods, the negative pressure method is slightly higher (less than 5%) than the immersion method, indicating that surface film thickness is less affected by the repair method.

When the urea in the repair solution completely decomposed, the surface film thickness by immersion method reaches 1.84 mm. Compared to the film thickness, which also used the immersion method by Qian et al. [35], the film thickness in this study is thicker. This is due to the concentration of urea and calcium ions in the repair solution. The highest concentration of the repair solution used by Qian et al. is 0.3 mol/L, and the corresponding surface film thickness is 0.289 mm; whereas the concentration of the repair solution used in this study is 1 mol/L, 3.33 times that used by Qian et al., the corresponding surface film thickness is 1.84 mm, 6.37 times that of Qian et al., indicating that SCIP is more advantageous for the growth of the surface film.

### 3.5. Average Infrared Temperature Increase

**Figure 15:**
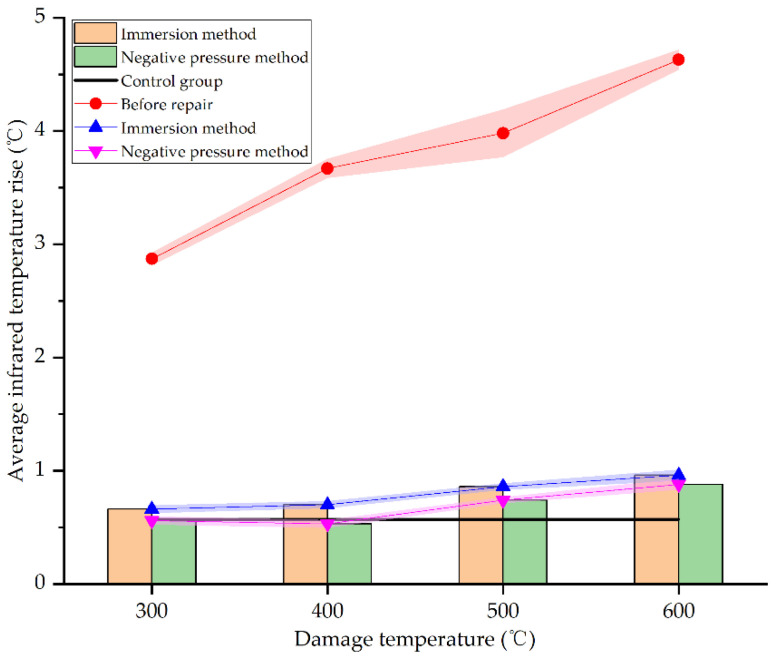
Average infrared temperature increase before and after repair by different methods and at different damage temperatures.

Figure 15 shows the average infrared temperature increase in concrete specimens before and after repair by different methods and at different damage temperatures.

As seen in Figure 15, the average infrared temperature increases of concrete specimens at different damage temperatures after repair all reduces significantly, very close to the undamaged specimens in the control group. At 300 °C, 400 °C, 500 °C, and 600 °C, the average infrared temperature increase in specimens repaired by the immersion method decreased by 77%, 80.93%, 78.39%, and 79.27%, respectively. This is consistent with the result obtained by Fan et al. [34]. However, the corresponding decline by negative pressure method is 80.49%, 85.56%, 81.41%, and 80.99%, respectively. The difference of average infrared temperature increase between different damage temperatures is less than 5% by the same repair method, indicating that damage temperature has little effect on the reduction of average infrared temperature increase. Furthermore, the average infrared temperature increase in the repaired specimens is very close to the undamaged specimens in the control group. This is because the decomposition of urea in the repair solution is relatively sufficient at 72 h, and carbonate ions chelate calcium ions to form sediments, which are attached to the surface of specimens. The surface film is flatter and denser than the concrete specimens damaged by high temperatures. The defects caused by high temperature are filled with sediments, making it difficult for external heat sources to accumulate heat on specimens’ surfaces, so the average infrared temperature increase reduces; this is consistent with the result of SEM analysis in Section 3.3. As for the two different repair methods, the average infrared temperature increase in concrete specimens repaired by the negative pressure method is slightly higher (less than 5%) than the immersion method. It signifies that the negative pressure method is more effective for the surface repair of high-temperature damage of concrete. However, there are only a few differences between the two methods. It can be seen from Section 3.4 and Section 3.5 that SICP has a good effect in surface repairing of high-temperature damage of concrete.

### 3.6. Water Absorption

**Figure 16:**
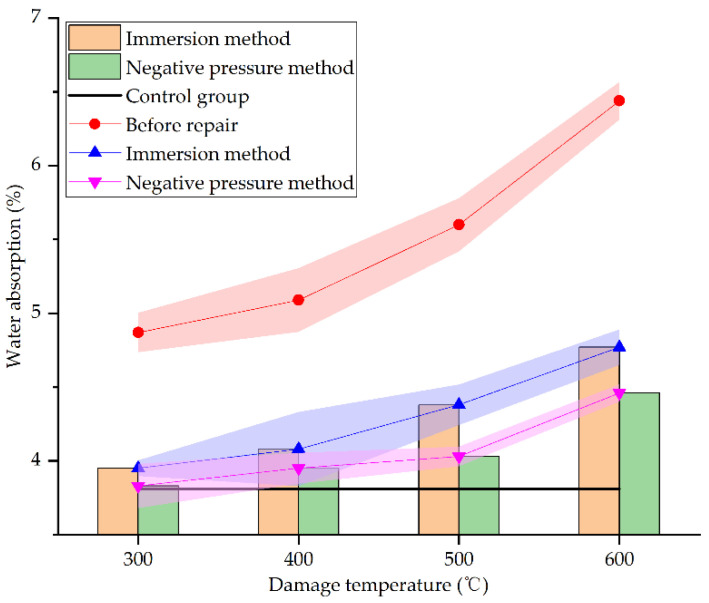
Water absorption before and after repair by different methods and at different damage temperatures.

Figure 16 shows the water absorption of concrete specimens before and after repair by different methods and at different damage temperatures.

From Figure 15, it can be seen that the water absorption of concrete specimens under different damage temperatures after repair reduces greatly. At 300 °C, 400 °C, 500 °C, and 600 °C, the water absorption of concrete specimens repaired by immersion method decreases by 18.89%, 19.84%, 21.79%, and 25.93%, respectively, whereas that by negative pressure method decreases by 21.36%, 22.4%, 28.04%, and 30.75%, respectively. When the damage temperature is between 300 °C and 400 °C, the water absorption of the two repair methods decreases slightly. When the damage temperature exceeds 400 °C, the water absorption of the two repair methods decreases greatly. This may be due to the more severe damage of specimens under higher temperatures. There will be more surface and internal defects in the specimens. The defects are even connected so that the repair solution is easier to enter the interior of specimens for repairing and filling, and the specimens are denser, which is consistent with the result of SEM analysis in Section 3.3. As for the two different repair methods, compared with the specimens before repair, the water absorption of specimens repaired by the negative pressure method decreases more, the absolute value of water absorption is also lower than that by immersion method, closer to the undamaged specimens. This is consistent with the result of SEM analysis in Section 3.3. It clearly shows that the negative pressure method is more effective in repairing high-temperature damage of concrete.

### 3.7. Compressive Strength

**Figure 17:**
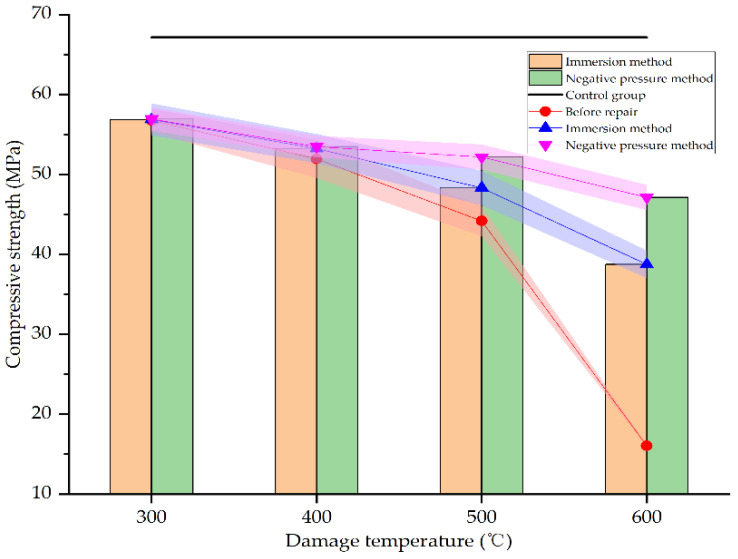
Compressive strength before and after repair by different methods and at different damage temperatures.

Figure 17 shows the compressive strength of concrete specimens before and after repair by different methods and at different damage temperatures.

As seen in Figure 17, after heating at different temperatures, the compressive strength of the specimens decreases in varying degrees. At 600 °C, the compressive strength is only 28% of the unheated specimens, reaching 16.04 MPa. However, after repair, the compressive strength improves in varying degrees. When the damage temperature is 300–400 °C, neither the immersion method nor the negative pressure method can significantly improve the compressive strength of damaged specimens. This is mainly due to the concrete damage is not serious in this temperature range. There are no obvious defects on the surface of the specimens. The rehydration of cement in the specimens makes the concrete specimens even denser. Whether immersion or negative pressure methods, the repair solution is difficult to enter the specimens. Only a layer of calcium carbonate film is formed on the surface of the specimens. The strength of this film is not high, and the bonding with the surface of specimens is weak. Therefore, the compressive strength of the specimens is not significantly improved compared with that before repair. When the damage temperature exceeds 400 °C, the compressive strength of concrete specimens is significantly improved after repair. At 500 °C and 600 °C, the compressive strength of the specimens repaired by immersion method increases by 9.37% and 141.58%, respectively, compared with the specimens before repair, and the compressive strength reaches 71.94% and 57.71% of the undamaged specimens, respectively. However, the compressive strength of the specimens repaired by the negative pressure method increases by 18.09% and 193.83%, respectively, compared with the specimens before repair, and reaches 77.68% and 70.19% of the undamaged specimens, respectively. This may be due to the more serious damage of the specimens at higher temperatures. There are more surface and internal defects in the specimens. The defects are even connected with each other so that the repair solution is easier to enter the interior of specimens for repairing and filling, and the specimens are denser. This is consistent with the result of SEM analysis in Section 3.3. The repair effect of the negative pressure method is more obvious than the immersion method since the negative pressure makes it easier for the repair solution to enter the specimens. It can be seen that the negative pressure method is more effective in repairing high-temperature damage to concrete.

## 4. Conclusions

This study extended the SICP technique to the field of high-temperature damage repair of concrete. The applicability of SICP on high-temperature damage repair of concrete was examined by a series of laboratory tests. Based on the results achieved during this study, the following conclusions are drawn:

(1) From the results of EDS and SEM, CaC_2_O_4_ and CaCO_3_ crystals are formed, CaCO_3_ crystals are larger, and the depose effect is better; these two kinds of crystals make the concrete denser.
(2) From the results of apparent characteristics, surface film thickness, and average infrared temperature increase, SICP has a good effect on the surface repair of high-temperature damage of concrete; the repair effect of the negative pressure method is slightly better than that of the immersion method.
(3) From the results of water absorption and compressive strength, SICP has a certain effect on the recovery of mechanical properties and durability of damaged concrete; the repair effect of the negative pressure method is obviously better than that of the immersion method.

This study revealed that the utilization of SICP in the repair of high-temperature damage of concrete is feasible theoretically. However, the actual application effect requires further examination. Firstly, repair method. The two methods in this study can be used in the laboratory, but they are difficult to apply in practice. Spraying, daubing, and painting are common in practical engineering. However, these methods were not used in this study; the repair effect of these methods needs to be examined. Secondly, repair effect at a lower temperature. The repair effect below 500 °C was not obvious. It needs further study to improve the repair effect when the temperature is not so high. Finally, regarding the internal repair, the surface repair effect was significant, but the internal repair effect has not been fully studied; thus, it needs to be further investigated. If these issues have been addressed, SICP will have a broad prospect in more fields in the near future.

## Figures and Tables

**Table 1:** Mix proportion of C50 concrete.

Cement (kg/m^3^)	Water (kg/m^3^)	Aggregate (kg/m^3^)	Sand (kg/m^3^)	Superplasticizer (kg/m^3^)	w/b	Slump (mm)
430	165	1130	720	1.15	0.38	152

**Table 2:** Specimens’ information for different tests.

Test	Sample Size (mm)	Repair Methods	Damage Temperature (°C)	Repair Time (h)
Apparent characteristics	40 × 40 × 40	Negative pressure method	600	72
EDS	White sediments from the surface of repaired specimen	Negative pressure method	600	72
SEM	Small blocks from the interior of repaired specimens	Immersion and negative pressure methods	600	72
Surface film thickness	40 × 40 × 40	Immersion and negative pressure methods	300, 400, 500, 600	24, 48, 72
Infrared thermal image	40 × 40 × 40	Immersion and negative pressure methods	300, 400, 500, 600	72
Water absorption	40 × 40 × 40	Immersion and negative pressure methods	300, 400, 500, 600	72
Compressive strength	40 × 40 × 40	Immersion and negative pressure methods	300, 400, 500, 600	72

**Table 3:** Element content of the white sediments.

Sample	C	O	Ca	Total
upper	28.08	57.82	14.10	100
bottom	18.82	64.03	17.14	100

## Data Availability

The data presented in this study are available upon request from the corresponding author.
